# An innovative linked Electronic Health Record derived data ecosystem for understanding the complexities of health and ageing.

**DOI:** 10.23889/ijpds.v9i5.2747

**Published:** 2024-09-18

**Authors:** Nadine Andrew, Richard Beare, Alison Carver, Tanya Ravipati, Emily Parker, Taya Collyer, David Ung, Velandai Srikanth

**Affiliations:** 1 Peninsula Clinical School, School of Translational Medicine, Monash University; 2 National Centre for Healthy Ageing; 3 Academic unit, Peninsula Health

## Objective

We describe the creation of an Electronic Health Record (EHR) derived linked data platform within the Australian National Centre for Healthy Ageing (NCHA).

## Approach

Our approach involved establishing a core set of EHR data suitable for research from the sole public health provider within a defined geographic region. Relevant items were selected based on published literature and consensus processes and curated within a specialised research data warehouse. Curation involved: data validation, quality assessment, internal linkage between episodes and data harmonisation/merging. An AI supported natural language processing pipeline was implemented to extract text-based data. Approvals were obtained from government agencies to link commonwealth claims data (primary care, medication, aged care, death) and state administrative hospital data, for all residents aged ≥60 years. Publicly available datasets were scoped for inclusion and collaborations established to incorporate local council data.

## Results

A core of 113 curated items for >900,000 people over 10 years have been established across 4 hospitals and 10 community/outpatient services. Linked state and commonwealth data were obtained for 179,089 residents aged >60 years (Jan 2010-May 2021, linkage accuracy 98.4%). Environmental (greenery, air pollution, walkability) and Census data have been incorporated for each neighbourhood (Statistical Area 1).  Data access, extraction and release has been tested through over 30 use-case projects in: dementia, residential aged care, medication use, homelessness, environmental impacts on health and ageing and health service redesign.

## Implications

The NCHA Data Platform provides an international exemplar for the use of linked EHR data in advancing population health research.

